# Quantification of Myoinositol in Serum by Electrochemical Detection with an Unmodified Screen-Printed Carbon Electrode

**DOI:** 10.1155/2022/3998338

**Published:** 2022-03-29

**Authors:** Xinrui Jin, Yuanqing Zhao, Xiujuan Gu, Min Zhong, Xin Kong, Guangrong Li, Gang Tian, Jinbo Liu

**Affiliations:** Department of Laboratory Medicine, Affiliated Hospital of Southwest Medical University, Luzhou, Sichuan 646000, China

## Abstract

Simple, rapid, and accurate detection of myoinositol (MI) concentration in blood is crucial in diagnosing polycystic ovary syndrome, neurological disorders, and cancer. A novel electrochemical detection (IED) method was established to quantify MI in human serum using a disposable unmodified screen-printed carbon electrode (SPCE) for the first time. MI was detected indirectly by the reaction product of myoinositol dehydrogenase (IDH) and cofactor *β*-nicotinamide adenine dinucleotide (NAD^+^). Good linear calibration curves were obtained at the concentration range from 5.0 *μ*M to 500.0 *μ*M (*R*^2^ = 0.9981) with the lower limits of detection (LOD) and quantification (LOQ) of 1.0 *μ*M and 2.5 *μ*M, respectively. Recoveries were calculated at three spiked concentrations, and the values were between 90.3 and 106%, with relative standard deviation values of 3.2–6.2% for intraday precision and 7.1–9.0% for interday precision. The SPCE-electrochemical biosensor is simple, accurate, and without modification, showing great potential for point-of-care testing (POCT) of serum MI in clinical samples.

## 1. Introduction

Inositol (cyclohexane-1, 2, 3, 4, 5, 6-hexol) is a polyalcohol under nine different stereoisomeric forms depending on the spatial orientation of its six hydroxyl groups [1]. In human and mammalian cells, myoinositol (MI) is the predominant physiological form (>99%), while the D-chiro-, scyllo-, epi-, neo-, and mucoinositols may be minor in quantity [2]. MI plays a crucial role in signal transduction, endoplasmic reticulum stress, and osmoregulation, as the precursor for inositol phosphates, phosphatidylinositol (PI), and phosphatidylinositol phosphate (PIP) lipids [3, 4]. In recent years, several studies have suggested that abnormalities in the MI metabolism have been implicated in reproductive issues [5], spinal cord defects [6], epilepsy [7], bipolar disorder [8], cancer [9], and neurological disorders [10]. Besides, orally administered MI can improve insulin sensitivity and reduce blood glucose concentration in human disorders associated with insulin resistance, including metabolic syndrome, polycystic ovary syndrome, and gestational diabetes [11–13]. Thus, the sensitive and accurate detection of MI in serum is of great importance for early disease diagnosis and treatment.

Currently, a few analytical methods have been developed to detect MI, including gas chromatographic-mass spectrometry (GC/MS) [14], liquid chromatography-mass spectrometry (LC/MS) [15], liquid chromatography-pulsed amperometric detection [16], and enzymatic cycling assay [17]. Although their sensitivity is high, HPLC, GC/MS, and LC/MS are time-consuming and require expensive instruments and highly skilled operators. Metabolite profiling by these conventional methods often requires long chromatographic run times ranging between 90 and 120 min per sample injection. Moreover, the enzymatic cycling assay is usually integrated with a biochemical analyzer in clinical with relatively large volume and nonportable equipment.

In comparison, electrochemical biosensors have become an attractive option for high throughput analysis with increased sensitivity, rapidity, and selectivity for data collection by using a small volume of the sample [18]. *β*-Nicotinamide adenine dinucleotide (NAD^+^) and its reduced form (NADH) that catalyzes electron transfer in metabolic reduction-oxidation (redox) reactions play major roles in the development of electrochemical enzyme biosensors [19–21]. However, the oxidation of NADH is slow and highly irreversible at bare electrode surfaces. As a consequence, it takes place only at high overpotentials (typically more than 1.0 V) and is followed by passivation and fouling of the electrode surface [22]. Recent approaches to the facilitated oxidation of NADH included the use of redox mediators, conductive polymers, and electrodes based on different forms of carbon which significantly decreased NADH overpotential [23, 24]. Moreover, in advance of technology, the screen-printed electrode (SPE) seems widely used in the electrochemical research field due to its simplicity, low cost, simple fabrication, fast response, disposability, and portability and can be used as a point-of-care (POC) device [25, 26]. We have recently reported on the development and extensive optimization of different electrochemical enzyme-based biosensors based on screen-printed carbon electrodes (SPCE) for the determination of several analytes in serum [19, 20]. The SPCE showed a very efficient electrocatalytic behavior toward oxidation of NADH at a low potential (approximately, 0.42 V).

In consequence, motivated by the fact that there is rapid growth in the demand for point-of-care tests (POCT), we have first developed a novel electrochemical biosensor using an enzymatic reaction couple with unmodified SPCE for detection of MI in serum samples. The serum MI was extracted from the biologic sample using the albumen precipitation method. Differential pulse voltammetry (DPV) of the electrochemical analytical technique was applied as the transduction mechanism of this biosensor. DPV used a linear sweep voltammetry with a series of fixed voltage pulses superimposed on the linear potential sweep [27]. The current was then determined directly before each potential change. Consequently, the effect of the charging current can be minimized, achieving in a higher sensitivity. DPV is frequently used in voltammetry-based techniques, not only due to its good sensitivity but also because of resolving power. This biosensor has several advantages, including, and most importantly, the fact that the electrode surface modification process is avoided under acceptable sensitivity. Under optimum conditions, the fabricated SPCE-electrochemical biosensor exhibited high sensitivity, wide dynamic range, excellent selectivity, and satisfactory reproducibility and stability for practical application.

## 2. Materials and Methods

### 2.1. Materials and Reagents

Myoinositol (MI) was purchased from Sigma Chemical (St. Louis, MO, USA). NAD^+^ and NADH were purchased from Roche Co. (Switzerland). IDH (the enzymatic activity of 250 U/ml) and myoinositol assay kits were bought from Megazyme International (Wicklow, Ireland). All chemicals were of analytical grade and used without further purification, and all aqueous solutions were prepared using ultrapure water from a Milli-Q system (Millipore, Billerica, MA, the United States). Electrochemical measurements were performed on a CHI660D electrochemical work station (CH Instruments, Shanghai Chenhua Instrument Corporation, Shanghai, China) with a portable commercial screen-printed carbon electrode (SPCE, Nanjing, China), which consisted of a carbon working electrode (3 mm diameter), a carbon auxiliary electrode, and an Ag/AgCl reference electrode.

### 2.2. Preparation of Standard Solutions and Serum Samples

Standard stoke solutions of NAD^+^ (40–220 mmol/L) and IDH (3000–7000 U/L) were prepared with ultrapure water and stored at −20°C. Serum MI determination was performed by the standard external method. Fifty clinical serum samples were mixed and used as a blank serum to establish and evaluate the method. The myoinositol assay kit (enzymatic UV method) was used for detection, and the determination was performed strictly according to the kit instructions. All serum samples were obtained from the Affiliated Hospital of Southwest Medical University (Luzhou, China) and stored at −80°C until use. The ethical committees approved this study of the Affiliated Hospital of Southwest Medical University. Fresh working standard solutions (50.0, 100.0, 200.0, 500.0, 1000.0, 2000.0, 4000.0, and 5000.0 *μ*mol/L) were prepared by diluting the stock solution (200 mmol/L) with ultrapure water to the required concentrations before use. MI calibration solutions were prepared by adding MI standard solution (100 *μ*L) into mixed blank human serum (900 *μ*L) to generate a concentration of 5.0, 10.0, 20.0, 50.0, 100.0, 200.0, 400.0, and 500.0 *μ*mol/L, respectively.

Sample preparation was accomplished through a protein precipitation procedure using acetonitrile. Spiked into centrifuge tubes was 120 *μ*L ml of serum, which was then mixed with 480 *μ*L of acetonitrile by vortex mixing for 30 s. The mixture was separated by centrifugation at 13300 rpm for 12 min at 4°C to remove protein. Subsequently, the supernatant was dried at 80°C under a nitrogen stream. The residue was dissolved in 40 *μ*L of 550 mmol/L glycine-NaOH buffer solution (pH = 10.3).

### 2.3. Electrochemical Measurement

Then, 5 *μ*L 160 mmol/L of NAD^+^ was added into 40 *μ*L of the residue in glycine-NaOH buffer solution in which MI reacted with NAD^+^ at the catalysis of 5 *μ*L of 4500 U/L IDH. After reaction for 20 min in centrifuge tubes at 35°C, 40 *μ*L of the mixed solution was put to the microcell on the SPCE, connected to a CHI660D electrochemical analyzer. Finally, the measurement of MI in serum was performed by DPV measurement with parameters of voltage (0.1 V to + 0.73 V), increment (0.005 V), amplitude (0.10 V), pulse width (0.05 s), the pulse period (0.2 s), quiet time (2 s), and sensitivity (1 × 10^−6^).

## 3. Results and Discussion

### 3.1. Principle of the Indirect Electrochemical Detection of MI

**Scheme 1:**
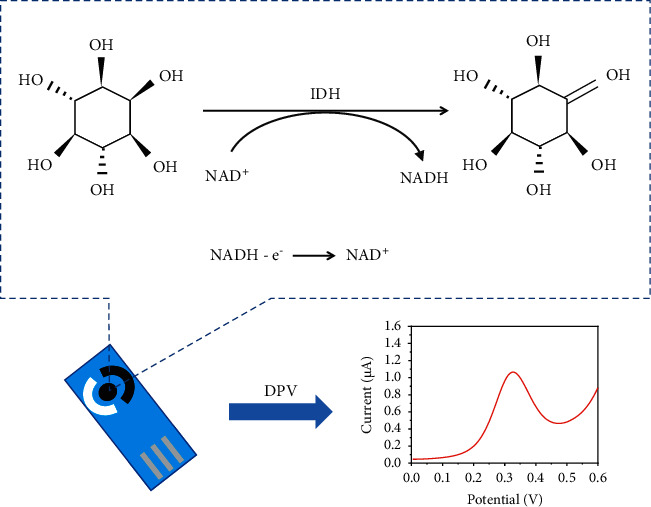
The mechanism of determination of MI by the electrochemical assay.

The principle of the enzymatic reaction has been clarified in our previous work [19]. As MI does not show electrochemical signals at SPCE, it was converted to 2, 4, 6/3, 5-pentahydroxycyclohexanone by IDH in the presence of NAD^+^, which was reduced to NADH. NADH could then be oxidized on the surface of SPCE, producing the detectable electrochemical signals. Consequently, the concentration of MI in human serum could be detected by these electrochemical signals collected by DPV, as shown in Scheme 1.

### 3.2. Indirect Electrochemical Behavior of MI

**Figure 1:**
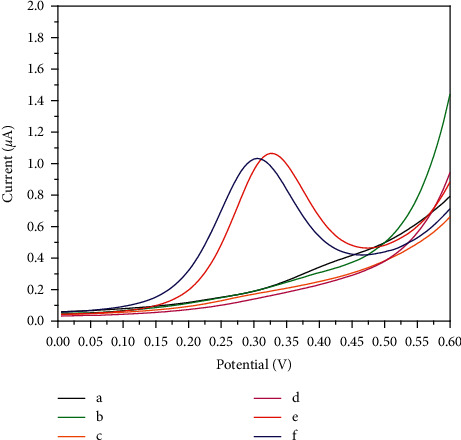
DPV curves of (a) 550 mmol/L glycine-NaOH buffer solution (pH 10.3), (b) 5 *μ*L of 160 mmol/L NAD^+^ and 5 *μ*L of 5000 U/L IDH in 40 *μ*L of 550 mmol/L glycine-NaOH buffer solution, (c) 5 *μ*L of 160 mmol/L NAD^+^ and 5 *μ*L of 2 mmol/L MI in 40 *μ*L of 550 mmol/L glycine-NaOH buffer solution, (d) 5 *μ*L of 5000 U/L IDH and 5 *μ*L of 2 mmol/L MI in 40 *μ*L of 550 mmol/L glycine-NaOH buffer solution, (e) 5 *μ*L of 160 mmol/L NAD^+^ and 5 *μ*L of 5000 U/L IDH and 5 *μ*L of 1 mmol/L MI in 40 *μ*L of 550 mmol/L glycine-NaOH buffer solution, and (f) 5 *μ*L of 500 *μ*mol/L NADH in 40 *μ*L of 550 mmol/L glycine-NaOH buffer solution.

To further testify whether MI could be detected indirectly on an unmodified SPCE, the DPV curves of different reagents were collected (Figure 1). No detectable DPV responses were observed in 550 mmol/L glycine-NaOH buffer solution (pH 10.3) that contains 160 mmol/L NAD^+^ and 5000 U/L IDH, or in the glycine-NaOH buffer solution that contains 160 mmol/L NAD^+^ and 5 *μ*L of 2 mmol/L MI, or in the glycine-NaOH buffer solution that contains 5000 U/L IDH and 5 *μ*L of 2 mmol/L MI (Figures 1(a)–1(d)). Whereas, elevated current responses were observed obviously when adding 2 mmol/L NADH into glycine-NaOH buffer solution. NADH was oxidized at the potential of about 0.32 V (Figure 1(f)). When MI was added into buffer solution containing of NAD^+^ and IDH, the oxidization peak was also detected. It indicated that with the catalysis of IDH, NAD^+^, and MI could react with each other, producing NADH which could be oxidized on the surface of the electrode and produced an electrochemical signal (Figure 1(e)). The result of the experiment matches well with the speculated indirect electrochemical reaction of MI.

### 3.3. Optimization of the Experimental Conditions for Electrochemical Detection

To achieve better electrochemical response and high detection sensitivity, several experimental parameters were optimized, including the ionic strength and pH of the supporting electrolyte, the concentrations of NAD^+^ and IDH, reaction time, and temperature.

#### 3.3.1. Optimization of the Ionic Strength and the pH of the Supporting Electrolyte

**Figure 2:**
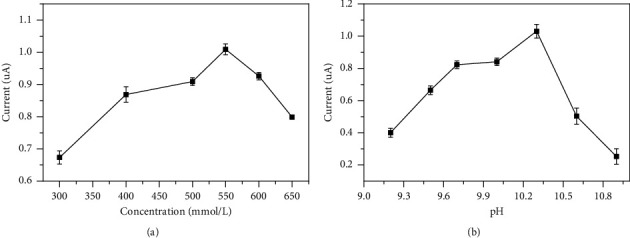
Effect of glycine-NaOH buffer solution concentration (a) and pH value of glycine-NaOH buffer solution (b).

The ionic strength and pH of the supporting electrolyte are essential parameters that cause a significant impact on the enzymatic reaction and the oxidation process of the product NADH on the electrode surface. IDH maintained a high enzyme activity under alkaline conditions [17]. It has been well established that NADH is stable under alkaline conditions, but is prone to autooxidation under acidic environments [28]. Thus, enzymatic reactions and oxidation of NADH are more likely to occur in alkaline media. The effect of ionic strength content and pH value of different buffer solutions on electrochemical determination is shown in in the supporting information Figure S1. It appears that glycine-NaOH buffer solution yielded the best results and was therefore used in all subsequent experiments below. Therefore, by utilizing DPV, the influence of glycine-NaOH buffer solution concentrations was investigated in the range of 300 mmol/L–650 mmol/L. The maximum value of the oxidation peak current occurred at 550 mmol/L and then decreased as glycine-NaOH buffer solution concentrations increased further, as shown in Figure 2(a). Also, in this study, different pH ranges were used from 9.5 to 10.9 to determine the most suitable pH for electrochemical detection. It was observed that as the pH of the reaction mixture increased, the oxidation current also increased and reach to its maximum at pH 10.3 (Figure 2(b)).

#### 3.3.2. Optimization of the Concentration of NAD^+^ and IDH

**Figure 3:**
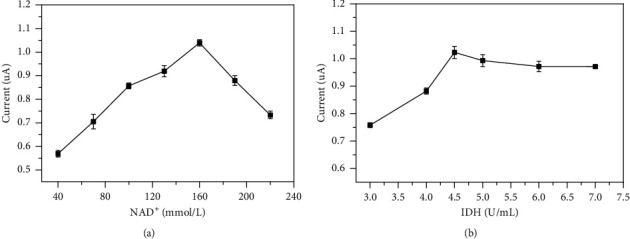
Effect of NAD^+^ concentration (a) and IDH concentration (b).

Under the catalysis of IDH, excess NAD^+^ should be used to ensure MI's complete reaction. The effect of the concentration of NAD^+^ (40 mmol/L–220 mmol/L) on the electrochemical signal was evaluated, as shown in Figure 3(a). To obtain high sensitivity, 160 mmol/L NAD^+^ was chosen for subsequent experiments. Moreover, the effect of enzymatic activity of IDH ranging from 3000 U/L to 7000 U/L was investigated, and the results are shown in Figure 3(b). Since 4500 U/L of IDH obtains the highest electrochemical signal, this concentration was selected for subsequent experiments.

#### 3.3.3. Optimization of Reaction Time and Temperature

**Figure 4:**
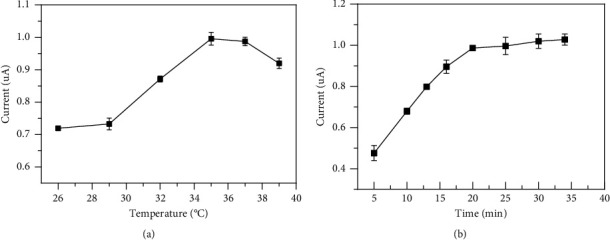
Effect of reaction temperature (a) and time (b).

The effect of reaction temperature on electrochemical detection was examined by utilizing DPV at different temperatures ranging from 26°C to 39°C (Figure 4(b)). The data showed that the peak current increased with increasing temperature, reached the maximum value at 35°C, and then decreased at temperatures over 35°C. This result may be attributed to the irreversible behavior (protein denaturation) involved in the process caused by high temperatures. Additionally, the reaction time was also investigated to guarantee a complete reaction. With the increasing time from 10 min to 41 min, the peak current suddenly enlarged and reached to a maximum value at 20 min and then slightly increased (Figure 4(a)).

### 3.4. Calibration Curve and Linearity

**Figure 5:**
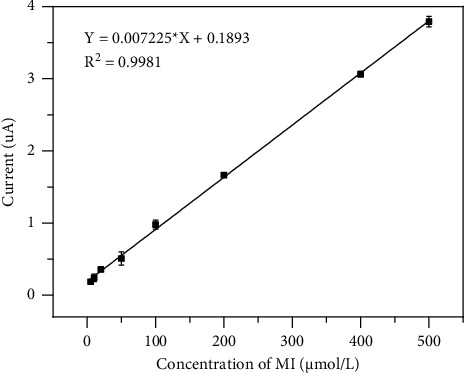
The calibration curve of MI by the established electrochemical method.

The calibration solutions were prepared by adding 100 *μ*L of MI standard solution into 900 *μ*L of the mixed blank human serum to generate a series of concentrations from 5.0 to 500.0 *μ*mol/L. The analytical performances of the sensor were evaluated by applying DPV. At the optimal experiment conditions, the electrochemical signals in both the standard calibration solution and blank serum were determined. The limits of detection and quantification (LOD and LOQ, respectively) were estimated as the lowest concentration of each analyte detected with a signal-to-noise ratio of 3 and 10, respectively, and <15% of relative standard deviations (RSDs) in three replicates. The LOD and LOQ were found to be 1.0 *μ*M and 2.5 *μ*M, respectively. The peak current of DPV was linear, with MI's concentration ranging from 5.0 to 500.0 *μ*mol/L. The calibration plot of the peak current versus the concentration of MI is shown in Figure 5 with equation *Y* = 0.007225^*∗*^*X* + 0.1893 (*R*^2^ = 0.9981, *n* = 3), where *Y* is the difference in the peak current of DPV between spiked serum and blank serum and *X* is the added concentrations of MI. The detection limit and linear range of the proposed method have been compared with that of the other previously reported methods for the determination of MI, as given in Table 1. It is evident that the proposed electrochemical method exhibits a good linear range with the low detection limit in the detection of serum MI, showing great application potential for MI detection from clinical complex fluids.

### 3.5. Precision and Recovery

The precision and recovery of the electrochemical method were examined by measuring the concentration of MI in 900 *μ*L blank serum before and after adding 100 *μ*L of MI standard solutions at high (200.0 *μ*mol/L), medium (100.0 *μ*mol/L), and low concentrations (10.0 *μ*mol/L). The intraday variation was determined by analyzing 5 replicate samples within one day, and the interday variation was examined on 5 consecutive days. Precisions were expressed by the relative standard deviation (RSD). As given in Table 2, the validation of precision ranged from 3.2% to 6.2% for intraday and 7.1% to 9.0% for interday, respectively. The detected concentration calculated the recovery by the established method divided by the added concentration of MI. The recoveries of different MI concentrations were from 96.6% to 106% for intraday and 90.3% to 103% for interday, respectively, achieving an acceptable recovery.

### 3.6. Interference Test

The interference experiment was performed by measuring clinical serum samples with different interfering substances (bilirubin and hemoglobin). The interference test was performed as reported in previous works by the authors [20]. The concentration of MI in serum before and after adding hemoglobin standards or bilirubin was defined as *X*_*C*_ and *X*_*T*_, respectively. The interference value (expressed as *X*_*T*_−*X*_*C*_) less than 1.96*S* showed little interference and was expressed by *N*. In contrast, the interference value more than 1.96*S* indicated significant interference and was expressed by *I*. The results showed significant interferences when hemoglobin concentration was over 4.0 mg/mL, and bilirubin was over 160 *μ*mol/L, as given in Table 3. Thus, in the established electrochemical method, it is crucial to avoid hemolysis for serum samples.

## 4. Conclusions

In conclusion, we have developed a novel indirect electrochemical method to quantify MI in serum with unmodified SPCE. Before analysis, the samples were prepared by protein precipitation to attenuate matrix effects in serum. Both the precisions and recoveries were sufficient to be used in a clinical setting. The short incubation time of 20 min suggests that our strategy will be a more advantageous method of quantification of MI in real samples than traditional chromatographic methods that normally takes 90–120 min for analysis. The proposed method is simple, rapid, of low cost, precise, accurate, and inexpensive regarding reagent consumption and the equipment involved.

## Figures and Tables

**Table 1:** Comparison of our research with other methods for MI detection in clinical samples.

Measurement methods	Sample	Linear range (*μ*mol/L)	LOD (*μ*mol/L)	Reference
Gas chromatography/mass spectrometry	Urine	1.4–1400	2.7	[14]
Amperometric determination using a CuS/GCE^1^	Urine	0.5–8.5	0.24	[29]
Liquid chromatography/mass spectrometry	Rat brain tissue homogenates	0.55–550	0.16	[15]
Inductively coupled plasma atomic emission spectrometry	Urine	0–10	0.1	[30]
High-performance liquid chromatography	Plasma	1.4–89	1.8	[31]
Enzymatic cycling method	Urine	Up to 2000	10	[17]
DPV using a unmodified screen-printed carbon electrode	Serum	5–500	1	This method

^1^CuS/GCE refers to a glassy carbon electrode modified with nanostructured copper sulfide.

**Table 2:** The precisions and recoveries of MI detected by the electrochemical method in human serum (*n* = 5).

Added concentration (*μ*mol/L)	Serum concentration (*μ*mol/L)	Measured concentration (mean ± SD, *μ*mol/L)	Precision (RSD, %)	Recovery (%)
Intraday				
10.0	4.5	14.20 ± 0.45	3.2	106
100	4.5	96.78 ± 5.22	5.4	96.7
200	4.5	197.58 ± 12.26	6.2	96.6

Interday				
10.0	4.5	14.84 ± 1.08	7.3	103
100	4.5	96.62 ± 8.63	9.0	90.3
200	4.5	198.20 ± 14.04	7.1	95.2

**Table 3:** The effect of hemoglobin and bilirubin on the determination of MI in serum by electrochemical assay.

Added hemoglobin (mg/mL)	Myoinositol (*μ*mol/L)			Added bilirubin (*μ*mol/L)	Myoinositol (*μ*mol/L)		
	Measured	*XT−XC*	1.96*S*		Measured	*XT−XC*	1.96*S*
0.0	150.65	—	10.16	0.0	150.65	—	10.16
2.0	149.16	*−*1.48	*N*	40.0	150.22	*−*0.42	*N*
4.0	147.27	*−*3.37	*N*	80.0	143.03	*−*7.61	*N*
8.0	123.90	*−*26.74	*I*	160.0	141.18	*−*9.46	*N*
12.0	—	—	*I*	180.0	138.46	*−*12.18	*I*
16.0	—	—	*I*	200.0	123.60	*−*27.04	*I*

*X*_*C*_, the concentration of MI in serum before adding hemoglobin/bilirubin; *X*_*T*_, the concentration of MI in serum after adding standards of hemoglobin/bilirubin; *N*, no significant interference; *I*, significant interference.

## Data Availability

The data used to support this study are included within the article.

## Abbreviations

CuS/GCE: Glassy carbon electrode modified with nanostructured copper sulfide
DPV: Differential pulse voltammetry
GC/MS: Gas chromatographic-mass spectrometry
HPLC: High-performance liquid chromatography
IDH: Myoinositol dehydrogenase
IED: Indirect electrochemical detection
LC/MS: Liquid chromatography-mass spectrometry
LOD: Limits of detection
LOQ: Limits of quantification
MI: Myoinositol
NAD+: *β*-Nicotinamide adenine dinucleotide
NADH: Reduced *β*-nicotinamide adenine dinucleotide
PI: Phosphatidylinositol
PIP: Phosphatidylinositol phosphate
POC: Point-of-care
POCT: Point-of-care tests
RSDs: Relative standard deviations
SPCE: Screen-printed carbon electrodes
SPE: Screen-printed electrode.

## Supplementary Materials

Supplementary Materials Figure S1. Effect of concentration (A-C) and pH value (D) of borax buffer, PBS buffer and glycine buffer on electrochemical determination.
