# Supplementary material for: Quantification of Myoinositol in Serum by Electrochemical Detection with an Unmodified Screen-Printed Carbon Electrode
Source: J Anal Methods Chem. 2022 Mar 29;2022:3998338. doi: 10.1155/2022/3998338 (PMC8983225; doi:10.1155/2022/3998338)
Supplement: Supplementary Materials — Figure S1. Effect of concentration (A-C) and pH value (D) of borax buffer, PBS buffer and glycine buffer on electrochemical determination. [file 3998338.f1.docx]

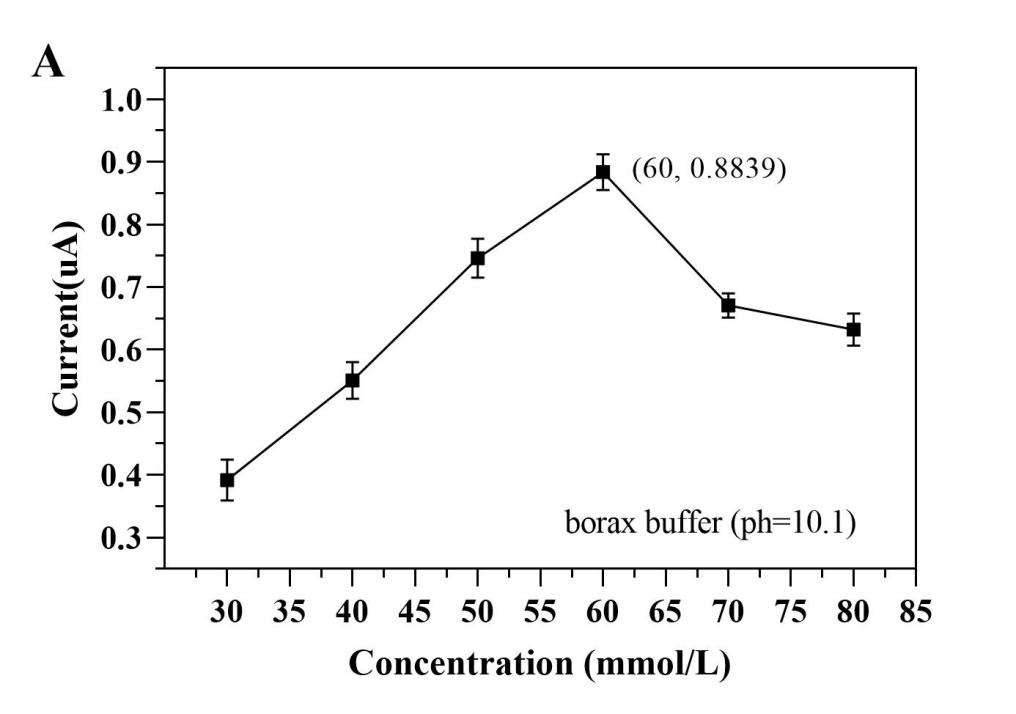

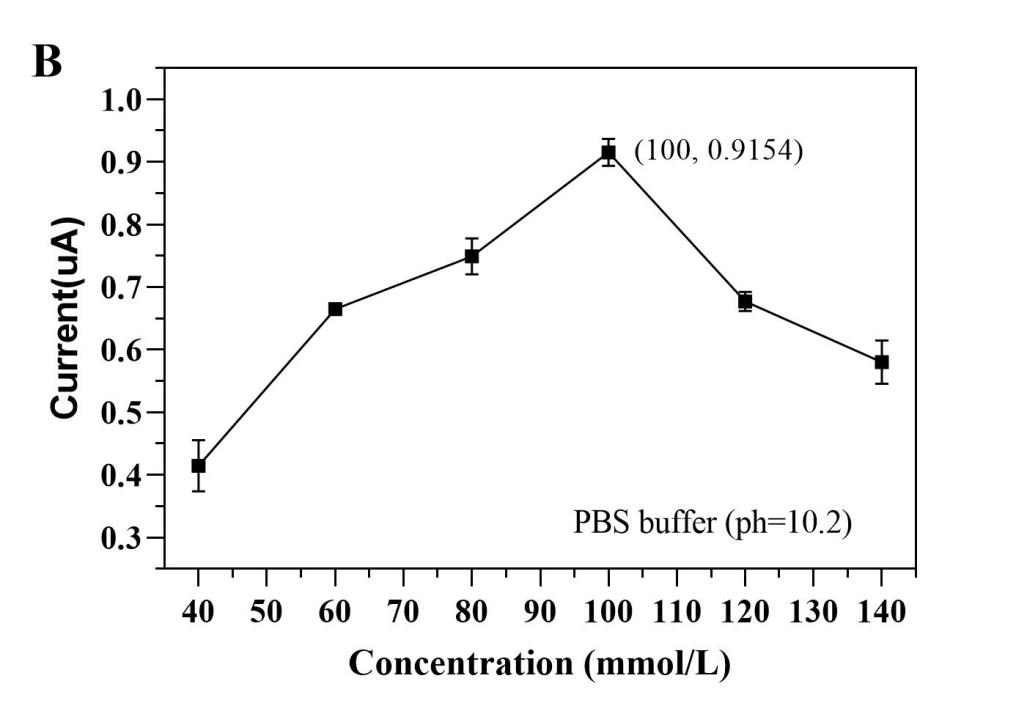

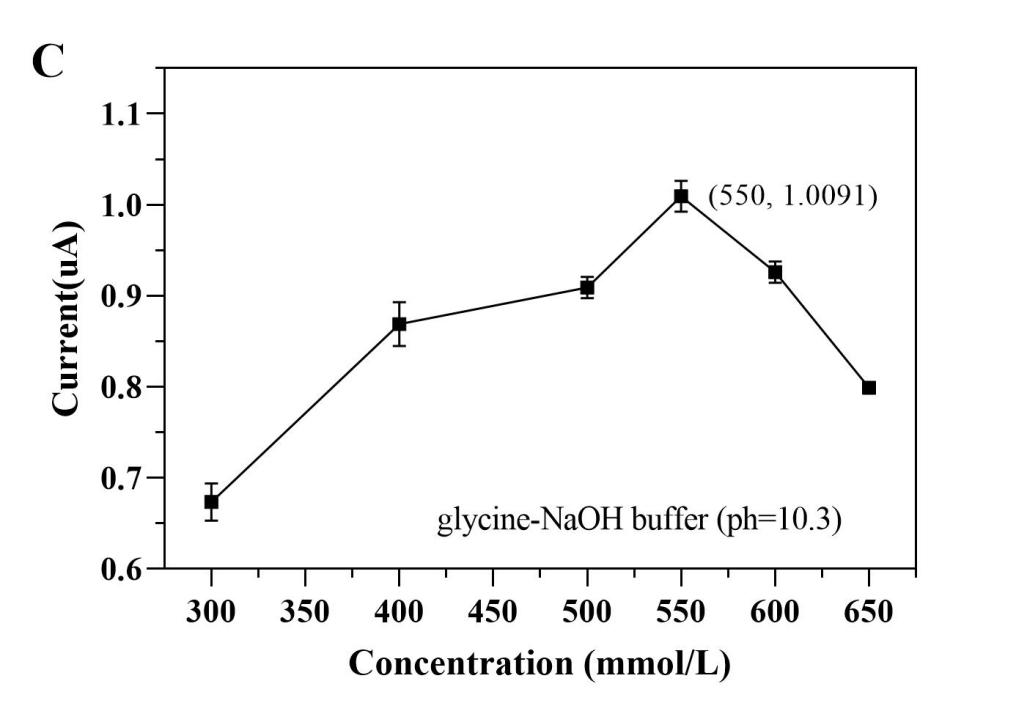

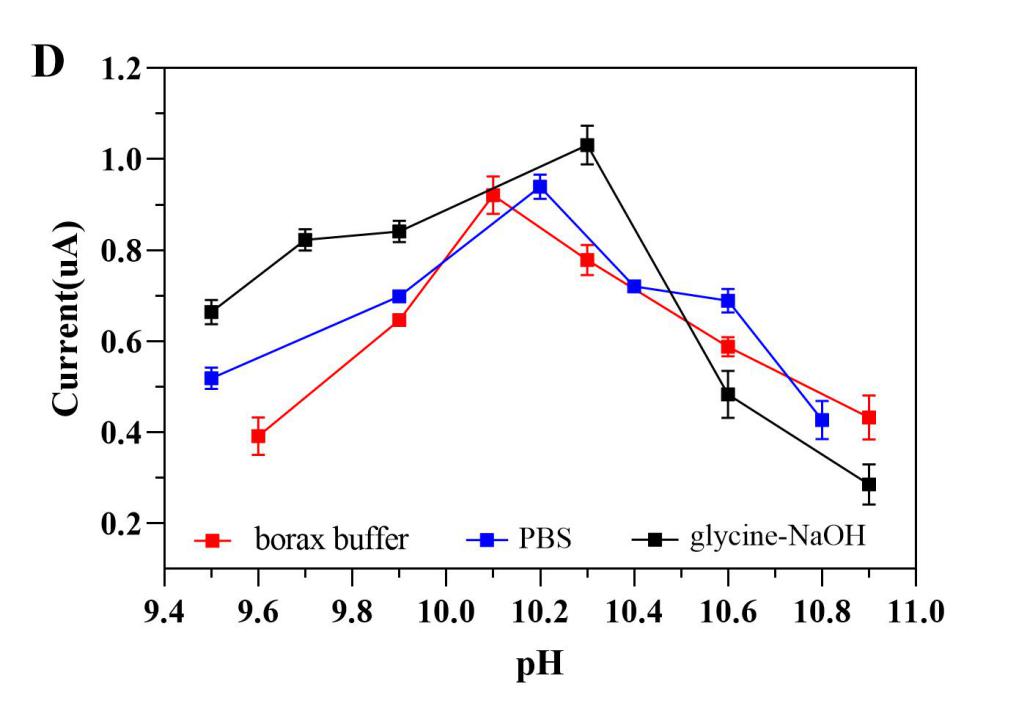


Figure S1. Effect of concentration (A-C) and pH value (D) of borax buffer, PBS buffer and glycine buffer on electrochemical determination.
